# Time to notice neurodiversity in eating disorder services: a three-year real-world analysis of autism, ADHD, and AuDHD

**DOI:** 10.3389/fpsyt.2026.1787957

**Published:** 2026-04-10

**Authors:** Lauren Makin, Karina Allen, Kate Tchanturia

**Affiliations:** 1 Institute of Psychiatry, Psychology and Neuroscience, Department of Psychological Medicine, King’s College London, London, United Kingdom; 2 National Institute for Health and Care Research (NIHR) Maudsley Biomedical Research Centre at South London and Maudsley NHS Foundation Trust and King’s College London, London, United Kingdom; 3 Department of Eating Disorders, South London and Maudsley NHS Foundation Trust, London, United Kingdom; 4 Department of Psychology, Ilia State University, Tbilisi, Georgia

**Keywords:** anorexia nervosa, attention-deficit/hyperactivity disorder, autism spectrum disorder, bulimia nervosa, comorbidity, eating disorders, functional impairment, psychological distress

## Abstract

**Introduction:**

Autism and ADHD frequently co-occur and each of them are overrepresented in clinical eating disorder (ED) services, where they are associated with longer treatment, poorer treatment experiences, and worse clinical outcomes. Separately, Autistic and ADHD patients with EDs present with greater ED psychopathology, anxiety, and depression. Autistic patients also present with poorer quality of life, increased suicide attempts, and greater functional difficulties. However, no study has directly compared patients with EDs who are both Autistic and ADHD (AuDHD) with Autistic-only, ADHD-only, or neurotypical patients.

**Method:**

This cross-sectional, observational study compared ED psychopathology (EDE-Q), psychological distress (CORE10), and work and social functioning (WSAS) across adult ED patients reporting suspected or known Autism, ADHD, both, or neither. No formal hypotheses were pre-specified. Data were three-years of routinely collected intake information from a specialist adult ED service. Patients were classified as Autistic or ADHD if they reported a diagnosis/suspicion at intake, or, for ADHD, if they reported ADHD medication.

**Results:**

Of 1,252 patients, 32 (2.6%) were classified as AuDHD, 45 (3.5%) as Autistic-only, and 81 (6.5%) as ADHD-only. Group differences were small but consistent. EDE-Q scores were highest in the ADHD-only group and lowest in the Neither group ((M = 4.24 *vs* M = 3.85, f=0.07). CORE10 and WSAS scores were highest in AuDHD and lowest in Neither (M = 24.16 *vs* M = 20.1, f=0.12; M = 26.56 *vs* M = 20.06, f=0.16).

**Discussion:**

Autistic and/or ADHD patients with EDs showed greater psychological distress, and poorer functioning, particularly when both were present. ADHD was particularly linked to increased ED psychopathology. Screening for neurodivergence in ED services may support person-centred care and improve outcomes.

## 1 Introduction

Autism and ADHD are common forms of Neurodivergence, characterised by differences in executive functioning, sensory processing, and communication (1). They frequently co-occur (2) and are both overrepresented in clinical eating disorder (ED) samples (3–6). A meta-analysis found that 29% of individuals with anorexia nervosa (AN), across child and adult samples, score above cut-off on gold standard Autism assessments (4). Autistic traits are also elevated in adults with bulimia nervosa (BN) (5). For ADHD, another meta-analysis has found that individuals with EDs are twice as likely to have ADHD compared to people without EDs, with particularly high prevalence in binge-type EDs such as BN (3). Reported prevalence estimates range from 3-35% in AN (3, 6, 7), 9-58% in BN (3, 5–7), and 29% in otherwise specified feeding or ED (OSFED) (6). In adult ED services, one study found that 31.3% of patients screened positively for ADHD (6).

Autism and ADHD are also linked to unique ED treatment needs (6, 8–16). Female Autistic patients with AN often present with greater anxiety and depression symptoms, across both adult and youth samples (11, 12), and higher ED psychopathology compared to non-autistic patients with AN (4, 13), though findings are mixed (12). Autistic patients with AN also demonstrate increased medication use (16), greater functional difficulties (11, 16), poorer quality of life (12) and higher rates of prior suicide attempts (13) compared to non-autistic patients with AN. Similarly, female ADHD patients, predominantly adults, with EDs show increased ED psychopathology (6, 14, 15) and increased anxiety and depression symptoms compared to non-ADHD patients with EDs (6, 15). This may be why Autism and ADHD are also linked to poorer outcomes (6, 8–16). In inpatient ED services, Autistic patients with AN may also often need longer or more intensive interventions compared to non-autistic peers (8). In adult female patients with EDs, ADHD has been associated with increased dropout rates and increased ED symptoms after treatment (9, 10). These differences may reflect greater clinical complexity and higher support needs in this group compared to non-autistic and non-ADHD individuals with EDs (6, 8, 11–16).

However, existing studies examine Autism and ADHD separately, despite their frequent co-occurrence; around 40% of Autistic individuals have co-occurring ADHD (2). This approach overlooks potential confounding, modifying, or unique effects associated with the combined presentation of Autism and ADHD (AuDHD). To our knowledge, no quantitative studies have examined AuDHD in patients with EDs. However, one qualitative study has explored the lived experiences and self-identified treatment needs of adults with BN or OSFED who reported known or suspected Autism and/or ADHD (17). Participants described how Autistic and ADHD traits contributed to both ED psychopathology and the types of support required during treatment (17).

The current exploratory study therefore aimed to examine associations between known or suspected Autism, ADHD, or both and ED psychopathology, psychological distress, work and social adjustment, and other clinical measures, in adult patients with EDs. This study will provide evidence using real-world clinical data to inform future confirmatory studies using larger datasets, such as those that will be provided by the UK Eating Disorders Clinical Research Network (EDCRN) (18).

## 2 Materials and methods

### 2.1 Data source

This exploratory cross-sectional secondary analysis used 33 months of routinely collected clinical data from the specialist adult ED service in South London and Maudsley (SLaM) NHS Foundation Trust, from December 2022 to August 2025. This service is not commissioned for binge eating disorder (BED) or avoidant/restrictive food intake disorder (ARFID). Data were collected via self-report intake questionnaires completed remotely prior to initial assessment using Microsoft Forms (see Appendix 1). Patients were informed that their de-identified questionnaire data would be used for routine clinical service evaluation and audit purposes. Patients could opt-out of completing measures if they chose. All patients who completed intake forms as part of their assessment were included, regardless of whether they were then accepted for treatment within the specialist ED service. Clinician-reported diagnoses were added manually to the datafile at a service level, before the removal of patient identifiers for data analysis. Diagnoses were made at assessment according to DSM-5 criteria (1). As no new data were collected and no changes were made to patient care, additional ethical approval and informed consent were not required in accordance with national regulations and institutional policies. Approval was obtained from the local National Health Service (NHS) Trust for routine clinical service evaluation.

### 2.2 Measures

#### 2.2.1 Known or suspected Autism or ADHD

Patients were classified as Autistic or ADHD if they reported suspected or diagnosed Autism or ADHD on their intake questionnaire. Most commonly this was recorded in response to items about mental health diagnoses or support needs. In addition, patients were classified as ADHD if they reported taking ADHD medications, including stimulants (Methylphenidate [Concerta XL, Xaggitin XL, Delmosart, Ritalin, Medikinet XL], Lisdexamfetamine [Elvanse, Vyvanse], Dexamfetamine, Modafinil) or the non-stimulant Atomoxetine. Patients were then grouped into four categories: AuDHD (both Autism and ADHD), Autism-only, ADHD-only, and Neither.

#### 2.2.2 Autistic traits (AQ-10)

Autism Spectrum Quotient, short version (AQ-10) is a 10-item self-report widely used measure to screen for Autism (19), with a clinical cut-off of 6 and above. Though frequently reported in ED research, its reliability in acute settings is debated due to low specificity (28%) and internal consistency (*a* = 0.64) (20, 21). Still, it is the only Autism screener currently recommended by NICE for adult clinical use and is one of the measures endorsed by EDCRN (18). In this study, the overall Cronbach’s alpha coefficient was acceptable (*a* = 0.72).

#### 2.2.3 ED psychopathology (EDE-Q)

Eating Disorder Examination Questionnaire (EDE-Q) is a 36-item self-report measure looking at ED symptomatology and behaviours over the last 28 days (22). The questionnaire provides scores across four subscales (dietary restraint, weight concern, shape concern, and eating concern), as well as a Global score, which reflects overall ED psychopathology. The EDE-Q is routinely administered at admission and discharge in most UK specialist ED services (18). In this study, the overall Cronbach’s alpha coefficient was excellent (*a* = 0.96).

#### 2.2.4 Psychological distress (CORE10)

Clinical Outcomes in Routine Evaluation (CORE-10) is a 10-item self-report measure looking at symptoms of psychological distress over the past week (23). It is commonly used in NHS mental health services and includes items about depression, anxiety, physical problems, trauma, general functioning, and risk to oneself. Scores can range from 0-40, with a clinical cut-off of 11. The CORE10 has been used previously in Autistic and ADHD populations (24, 25). In this study, the overall Cronbach’s alpha coefficient was acceptable (*a* = 0.70).

#### 2.2.5 Work and social functioning (WSAS)

Work and Social Adjustment Scale (WSAS) is a 5-item self-report measure assessing the extent of difficulty in the following domains: home management, ability to work, both social and private leisure, and ability to form and maintain close relationships (26). Each domain is scored on a 9-point Likert scale, ranging from 0 (no difficulty) to 8 (very severe difficulty). The maximum total score is 40, with a clinical cut-off of 20 and above. WSAS is widely reported in many mental health conditions, including in ED populations (11, 27, 28). In this study, the overall Cronbach’s alpha coefficient was good (*a* = 0.86).

#### 2.2.6 Demographic information

In addition to reporting on other mental and physical health diagnoses, patients self-reported their gender identity, ethnicity, educational level, work status, living circumstances, medications, smoking status, alcohol use, past suicide attempts, ED duration, weight/height (which were also measured at assessment), prior ED treatment, absences from school or work due to an ED, and any support needs.

### 2.3 Data analysis

Data were primarily cleaned manually in Excel, but duplicate entries were removed automatically. Descriptive statistics for each group were generated in IBM SPSS Statistics for Mac, version 31. Missing data were generally low, except for ED diagnoses which were absent in 20.93% of cases because they must be entered manually by clinicians after intake, unlike the rest of the questions which are self-report, and support needs as many cases left this section blank. Within the Neither group, 6.12% of cases had missing age data, 7.31% of cases had missing weight trend data, 5.12% had missing smoking data, and 5.30% had missing alcohol consumption data (in part, because these questions are not mandatory). For all other measures, missingness was <5% (see **Supplementary Table S1**).

This was an exploratory, descriptive study; analyses were conducted to examine potential differences between groups rather than to test pre-specified hypotheses. Standardised clinical cut-offs were used to aid interpretation, alongside effect sizes. Effect sizes for categorical outcomes were estimated using Cramer’s *V*, and for continuous outcomes using Cohen’s *f*. Cramer’s *V* was interpreted as small≈0.1, medium≈0.3, and large≈0.5 (29). Cohen’s *f* was interpreted as very small≈0.005, small≈0.10, medium≈0.20-0.31, large≈0.32-0.40, very large≈0.44-0.60, and huge≈1.0 (29–31). Given small cell sizes (0 or <5) in some categories, estimates of Cramer’s *V* should be interpreted with caution.

## 3 Results

### 3.1 Demographics

A total of 1,252 patients were included in this study. Of these, 32 (2.6%) reported potential Autism and ADHD or were on ADHD medication (AuDHD), 45 (3.5%) reported potential Autism only (Autism-only), and 81 (6.5%) reported potential ADHD only or were on ADHD medication (ADHD-only). The remaining 1,094 (87.4%) patients did not report Autism or ADHD (Neither). Most cases were female (88.9%), heterosexual (74.9%), and white (74.8%). All cases were adults (*M* = 29.23 years, *S.D* = 9.94). Group differences in age were small (*f* = 0.07); mean age was 31.50 years (*S.D* = 9.37) in the AuDHD group, 27.29 years (*S.D* = 8.83) in the Autistic-only group, 30.94 years (*S.D* = 9.22) in the ADHD-only group, and 29.11 years (*S.D* = 10.04) in the Neither group. Participants’ highest level of education was most commonly a university degree (31.0%), upper-secondary or vocational qualification (A level/NVQ; 21.2%), postgraduate degree (17.2%), lower-secondary qualification (O level/GCSE; 11.7%), other secondary vocational diplomas (BTEC; 11.9%), and no formal qualifications (2.9%). Demographic differences across groups were small (Cramer’s *V* = 0.05-0.11; see **Supplementary Table S2**), with lower rates of heterosexuality observed in the AuDHD (55.17%), Autism-only (52.63%), and ADHD-only (63.89%) groups compared to the Neither group (76.38%).

### 3.2 ED presentation

**Figure 1:**
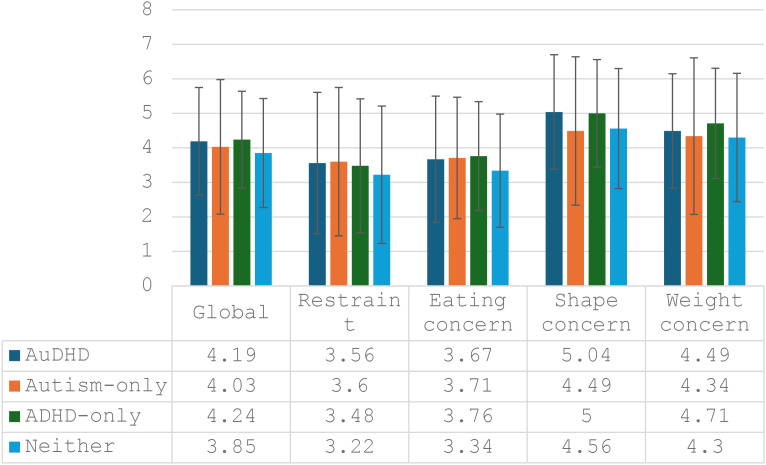
EDE-Q global and subscale scores (restraint, eating concern, shape concern, and weight concern) across the four groups (AuDHD, dark blue; Autism-only, orange; ADHD-only, green; and Neither, light blue). Error bars show standard deviation.

Most of the sample had anorexia nervosa (AN; 32.54%), bulimia nervosa (BN; 25.62%) or otherwise specified feeding or ED (OSFED; 33.37%), were treated in outpatient services (93.13%), and reported stable weight in the three months prior to assessment (41.83%; see **Supplementary Table S2**). Group differences were small (Cramer’s *V* = 0.04-0.07). AN was underrepresented and BN overrepresented in the ADHD-only group (29.69% for each); BED was underrepresented in the Autism-only group (0.00%); and ARFID was overrepresented in both the Autism-only (13.33%) and ADHD-only groups (9.38%). There was a very small difference in EDE-Q global scores across groups (*f* = 0.07; see **Supplementary Table S3**), with the highest average score in the ADHD-only group (*M* = 4.24), and lowest in the Neither group (*M* = 3.85). Subscale comparisons were not performed; raw scores are shown in **Figure 1**.

### 3.3 Autism, ADHD, and support needs

Overall, mean scores for AQ-10 were 4.44 (*S.D* = 2.51). A large difference was found in AQ-10 scores across groups (*f* = 0.34). Both the AuDHD (*M* = 7.84) and Autism-only groups (*M* = 6.73) exceeded the cut-off threshold of six, whereas ADHD-only (*M* = 5.35) and Neither (*M* = 4.17; see **Supplementary Figure S1**) remained below it. Similarly, a medium group difference was observed for family history of Autism (Cramer’s *V* = 0.30; see **Supplementary Table S4**), with higher rates in AuDHD and Autism-only groups (73.3-75.0%) compared to ADHD-only and Neither (21.8-33.3%). Among patients, 28.1% (*n* = 9) of the AuDHD group and 34.6% (*n* = 28) of the ADHD-only group reported taking ADHD medication. There were small group differences in support needs (Cramer’s *V* = 0.06-0.11; see **Supplementary Table S4**) and AuDHD and Autism-only patients (31.1-37.5%) were more likely to request verbal support than ADHD-only or Neither groups (16.8-17.3%). Many specifically reported (suspected or diagnosed) Autism or ADHD here and made specific support requests in relation to these (see **Table 1**). Dyslexia was the other most frequently mentioned condition in relation to support needs (*n* = 7).

**Table 1:** Summary of support requests described by patients as relating to their Autism or ADHD.

Neurotype	Self-reported support needs
Autism	Uses alternative communication methods; information processing difficulties so needs extra time and clear and concise instructions; struggles with new/unfamiliar situations and benefits from predictability; experiences sensory sensitivities/overwhelm; may struggle with social communication and eye contact.
ADHD	Email communication preferred as difficulty processing and retaining new information; easily overwhelmed by sensory input; needs breaks, extra time, and support with focus, organisation, and time management.

### 3.4 Psychological distress and work and social functioning

**Figure 2:**
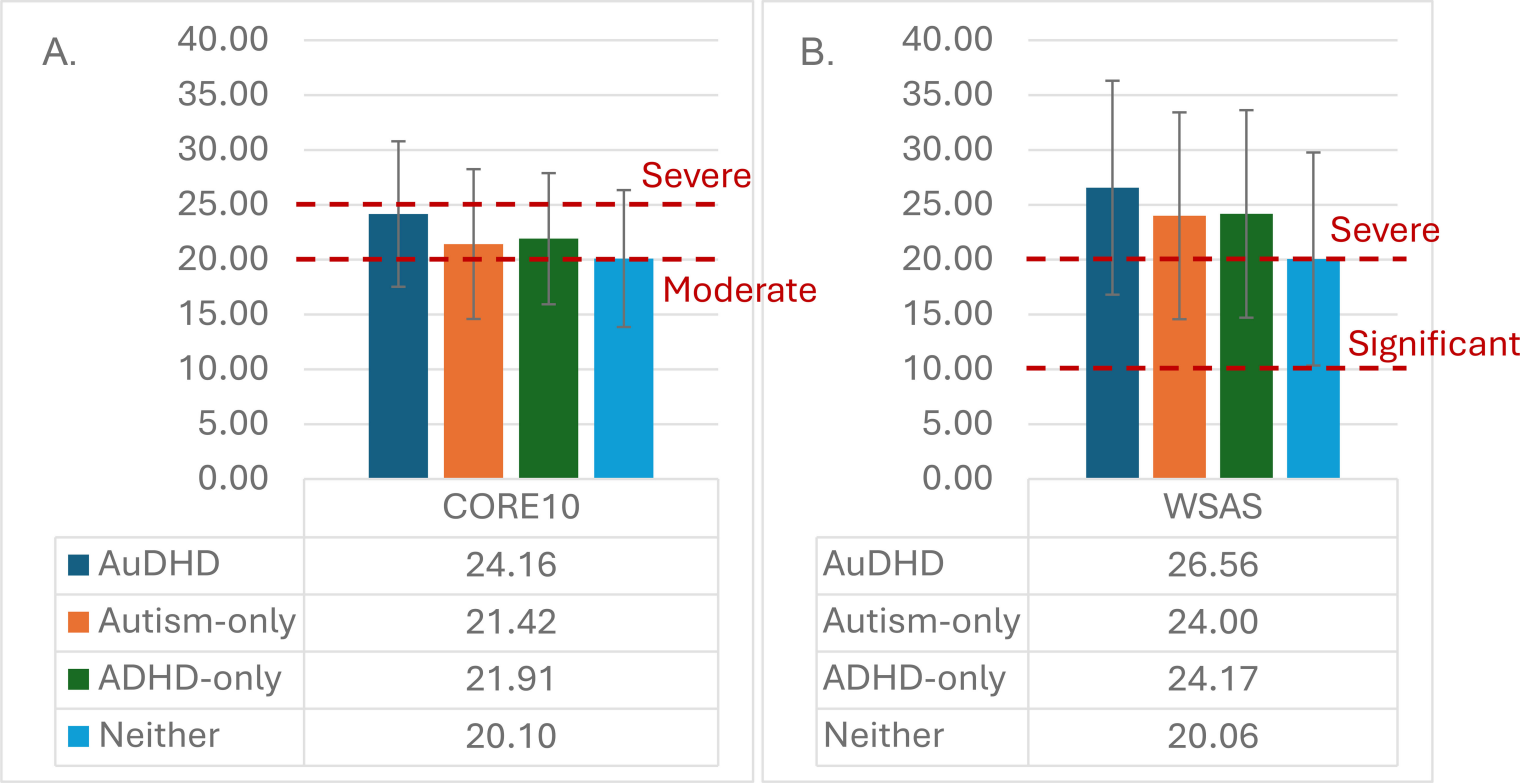
**(A)** CORE-10 scores and **(B)** WSAS scores across the four groups. In panel **(a)**, dotted red lines indicate the cut-offs for moderate and severe symptoms; in panel **(b)**, dotted red lines indicate the cut-offs for significant and severe difficulty. Error bars show standard deviation.

There were small differences in absences from work and suicide attempts (Cramer’s *V* = 0.15-0.18) with higher absences from work in AuDHD (56.00%), Autism-only (48.28%), and ADHD-only (42.19%) compared to Neither (21.61%) and higher rates of suicide attempt in AuDHD (56.25%), Autism-only (42.22%), and ADHD-only (35.80%) compared to Neither (24.64%). There were smaller differences in absence from school, alcohol consumption, and smoking between the four groups (Cramer’s *V* = 0.07-0.10). Overall, mean scores for CORE10 were 20.37 (*S.D* = 6.29), and for WSAS were 20.63 (*S.D = *9.80). There was a small difference (*f* = 0.12) in CORE10 scores across all four groups, with the highest scores for the AuDHD group (*M* = 24.16), and lowest for the Neither group (*M* = 20.10; see **Figure 2a**); all between the moderate and severe range. There was also a small difference (*f* = 0.16) in WSAS scores across all four groups, with the highest scores for the AuDHD group (*M* = 26.56), and lowest for the Neither group (*M* = 20.06; see **Figure 2b**); all in the severe range.

## 4 Discussion

This naturalistic, descriptive study examined associations between known or suspected Autism, ADHD, or both, and ED psychopathology, psychological distress, and work and social functioning in a large sample of adult patients in a specialist ED service. Patients reporting suspected or known Autism or ADHD exhibited higher ED psychopathology, psychological distress, and lower work and social functioning than those not reporting Autism or ADHD. ED psychopathology was highest in ADHD-only patients. In contrast, psychological distress was highest, and work and social functioning lowest, in patients reporting co-occurring Autism and ADHD, suggesting unique effects for these outcomes. Findings suggest that Autistic and ADHD patients with EDs present with greater clinical complexity and may require tailored, personalised treatment support.

### 4.1 ED psychopathology

Interestingly, co-occurring Autism and ADHD did not produce unique effects on ED psychopathology; ADHD-only patients showed the highest global EDE-Q scores. This aligns with previous findings from a non-clinical sample in which both Autistic and ADHD traits were associated with ED psychopathology (32). However, Thomas and colleagues (32) reported that once anxiety and depression were controlled for, Autistic traits were no longer significantly associated with ED psychopathology, whereas ADHD traits remained a significant predictor. This suggests that ADHD may have a more direct influence on ED psychopathology, while Autism may exert its effects primarily through co-occurring anxiety and depression. Another possibility is that Autism and ADHD may influence ED behaviours differently: Autism is more strongly linked to restrictive eating (4, 17, 33, 34), which can temporarily reduce ED cognitions when weight control goals are met, whereas ADHD is associated with impulsive, binge-purge behaviours (3, 17, 33) that perpetuate ED cognitions.

Additional prior studies have also found links between potential ADHD and higher ED psychopathology (14, 15). Fernandez-Aranda and colleagues (14) found that in female patients with EDs, ADHD traits were significantly associated with ED psychopathology, particularly interoceptive difficulties. Similarly, Seitz and colleagues (15) reported that female patients with BN had increased ED psychopathology if they had probable ADHD. ADHD may heighten risk for binge eating and weight gain (5, 17, 33, 35), contributing to greater body dissatisfaction and shape concerns. Svedlund and colleagues (6) found probable ADHD was positively associated with loss of control and binge eating in patients with EDs, and prevalence estimates of ADHD are higher in binge-type EDs (9-67%) than restrictive subtypes (3-35%) (3, 5, 6). Broader difficulties and low self-esteem associated with ADHD may further increase vulnerability to negative cognitions, reinforcing eating, weight, and shape concerns (36). Equally, it is worth noting that ED behaviours such as bingeing or purging may inflate ADHD traits, due to the effects of these behaviours on emotion, cognition and behaviour, and some evidence shows modest reductions in ADHD symptoms following ED recovery (37).

Autistic patients also had elevated ED psychopathology, largely consistent with previous work (4, 13). A meta-analysis of seven previous studies investigating the relationship between Autistic traits (AQ specifically) and ED psychopathology in patients with AN (38–44), found a weak positive correlation between the two, and showed no evidence of publication bias (4). Similarly, Zhang and colleagues (13) reported elevated ED psychopathology in patients with AN and confirmed Autism diagnoses, including a 0.6-point higher EDE-Q global score. Restrictive eating in Autistic patients may stem from exteroceptive hypersensitivity causing food aversions, interoceptive hyposensitivity reducing internal sensitivity to hunger cues, monotropism reinforcing strict food rules and routines, and emotional overwhelm or alexithymia (17, 33, 34). Consequently, EDE-Q might even underestimate the nature or severity of ED symptoms in Autistic individuals, as it may miss sensory-related food avoidance, although there is also a risk that this measure pathologizes Autistic eating behaviours that are not necessarily harmful (45). Eating, weight, and shape concerns may also arise in Autistic individuals from social-communication differences, as non-Autistic people may misunderstand or judge these differences (34, 46–48). This can cause significant emotional and psychosocial distress for Autistic people, contributing to low self-esteem and efforts to ‘fit in’ or belong (34, 46–48).Overall, these mechanisms likely contribute to the overrepresentation of Autism in restrictive EDs, particularly AN, where ~29% of individuals meet diagnostic criteria for Autism as determined by gold-standard semi-structured clinical observations (4). However, Autistic traits can also contribute to binge-type EDs and overeating as well (17, 33, 49), although this has been understudied (5, 35).

### 4.2 Psychological distress

Patients reporting suspected or known Autism or ADHD also showed greater psychological distress (CORE-10), with the co-occurring group most affected. Previous work in non-ED populations has shown that both Autistic and ADHD traits are positively associated with CORE10 scores (24, 25). However, to our knowledge, this is the first study to apply a broad measure of psychological distress to Autistic and ADHD patients with EDs. Risk to self was also elevated in the current study, as has previously been found in patients with AN and a confirmed Autism diagnosis (13).

Despite the lack of previous research looking at general psychological distress, prior research in ED populations does demonstrate elevated anxiety and depression in Autistic and ADHD patients (6, 11, 12, 15). Autistic traits have been linked with higher anxiety, depression, and quality of life in patients with AN (11, 12), while ADHD traits have similarly been associated with greater anxiety and depression in both mixed ED and BN-only samples (6, 15). However, none of these studies directly compared Autism and ADHD, which is a key contribution of the current study.

Both Autism and ADHD confer overlapping and distinct risks for anxiety, depression, trauma, physical difficulties, and risk to self (35, 50–58), which may compound when co-occurring. Mechanisms connecting Autism to these outcomes may include sensory sensitivities, intolerance of uncertainties, and stigma, discrimination, and social exclusion (59–63), and for ADHD, may include executive dysfunction and increased daily stressors (64, 65). Both Autistic and ADHD individuals may also experience sleep problems (52, 66) and emotional regulation difficulties (67, 68).

### 4.3 Work and social functioning

Work and social functioning, measured via the WSAS and partly captured by the CORE10, was lower in suspected or known Autistic and ADHD patients, with unique effects in the co-occurring group. In addition, Autistic and ADHD patients were more likely to report absences from work or school due to their ED. This aligns with findings by Tchanturia and colleagues (11), who reported that increased Autistic traits correlated with lower work and social functioning in patients with AN. Similarly, Nazar and colleagues (16) found that adolescents with (atypical-/)AN and Autistic traits showed greater emotional and behavioural difficulties (SDQ). This could be due to sensory overload in daily environments (69), challenges with social communication with non-Autistic individuals (70), and monotropism affecting engagement at school/work (71).

To our knowledge, no studies have previously considered work and social functioning in ADHD patients with EDs. However, research in non-ED populations consistently demonstrates that ADHD adults experience significant difficulties across academic, occupational, and social domains, including underachievement, unemployment, absenteeism, and difficulties sustaining relationships (72). These difficulties are thought to stem primarily from executive functioning challenges, such as inattention, time-blindness, impulsivity, and disorganisation (72). However, they may also be amplified by anxiety and depression, which are also known to worsen functional outcomes (73). ED populations have also previously demonstrated lower WSAS scores compared to controls, with intermediate scores for recovered ED patients (27, 28), but neither Autism nor ADHD was measured in these studies.

Autistic patients were also more likely to report verbal support needs than non-Autistic patients, and described requiring clear, concise communication, extra time to process information, and a predictable environment due to sensory sensitivities and social communication difficulties. This aligns with previous findings that Autistic patients with EDs prefer adjustments to dietary guidance, meal plans, and therapeutic environments to accommodate sensory needs; flexible communication options, allowing additional time for verbal processing, and use of clear, concrete language; and consistent and predictable appointments and routines during treatment (17, 33, 34). ADHD patients also described benefiting from email communication to support information processing and understanding, as they described becoming easily overwhelmed by sensory input and new information; they requested breaks, additional time, and support with focus, organisation, and time management. Again, this aligned with previous findings that ADHD patients with EDs valued treatment that helped them develop structure and routine and that accommodated their sensory and communication needs (17, 33).

### 4.4 Clinical implications and support needs

These findings highlight the increased needs of Autistic, ADHD, and AuDHD patients with EDs. In particular, these difficulties were around increased ED psychopathology, psychological distress, and work and social functioning difficulties. Thus, these findings highlight the need for individualized support for Autistic and ADHD patients with EDs, as has been called for previously by Autistic and ADHD individuals (17, 74–78). It is important to involve the population of interest when making decisions about tailoring treatment, so incorporating qualitative data from Neurodivergent individuals could offer valuable insights when determining how to best adapt treatment.

Support needs described by the patients on their intake forms in the current study match with those in prior studies. Autistic patients reiterated the need for alternative communication methods, for clear and concise instructions, and for more time to process information (33, 74, 76, 77, 79). They also described wanting predictability, and needing sensory needs to be managed to enable eating (17, 33, 74, 76, 79). ADHD patients also described needing breaks and extra time, and support with focus, organisation, and time management (17, 33). Thus, individualised support in ED services for Autistic and ADHD patients could include support in communication, managing sensory needs to enable eating, reduce overwhelm, and improve interoception, as suggested by these populations themselves (17, 33, 74, 76, 79).

Helpful options include sensory workshops (80), sensory toolkits, interoception-based interventions like ADIE (Aligning Dimensions of Interoceptive Experience; improves interoceptive awareness and reduces anxiety in Autistic adults (81)), and sensory adaptions to dietary guidance, meal plans, and therapeutic environments. Practical strategies (e.g. fidget toys, weighted blankets, or textured foods) and ‘dopamine menus’ (82) to regulate stimulation needs may also be helpful (33). Autistic and ADHD adults with binge eating additionally recommend emotion wheels, structured meals, and alternative self-soothing strategies (33). Additionally, a pre-requisite for such support is identifying Autism and ADHD, for example, via routine use of screening tools like the AQ-10 (83), the Adult ADHD Self-Report Screener Scale (ASRS) (84), and the Glasgow Sensory Questionnaire (GSQ-14) (85).

Clinical adaptations such as the PEACE pathway (86), which includes Autism and sensory screening, staff training, environmental adjustments, communication passports, and sensory workshops (87–89), demonstrate that Autism-informed adaptations can improve outcomes and reduce treatment duration, receiving positive feedback from patients and clinicians and saving approximately £22,837 per patient (90, 91). Similar approaches may benefit ADHD, especially due to the overlap in treatment needs of Autistic and ADHD patients (17, 33). However, most existing evidence focuses on Autism rather than ADHD, and only a small number of studies have explored ADHD patients’ own views on their treatment needs (17, 33).

These findings also suggest that some patients may additionally benefit from referral to broader support pathways, such as community mental health or Autism/ADHD services, to address co-occurring psychological distress and difficulties in work and social functioning.

### 4.5 Strengths and limitations

This study is the first to examine both Autism and ADHD within the same clinical ED sample, allowing for comparisons and exploration of potential unique effects. It also used routinely collected data from a large specialist ED service, capturing patterns and complexities reflective of actual clinical practice, rather than highly controlled research settings. The sample was predominantly female, white, and heterosexual, consistent with wider ED literature and patterns of service access (92, 93). Notably, heterosexuality was lower in the Autistic and ADHD groups, aligning with prior research, particularly on women (94). Despite relying partially on self-reported suspected diagnoses, the Autistic group showed elevated AQ-10 scores and higher family history of Autism, providing convergent validity. Similarly, these measures were also slightly elevated in the ADHD group, highlighting the overlapping symptomatology and heritability between Autism and ADHD (95–97).

However, analyses were descriptive and cross-sectional, limiting causal inference. Because the data are cross-sectional, relationships between neurodivergence, ED psychopathology, psychological distress, and work and social functioning cannot be interpreted causally. Further confirmatory and longitudinal studies are needed to validate these findings. Clinical datasets are also prone to missing information, and certain variables, such as ED diagnosis and weight trends had substantial missingness, which could bias results.

As this study relied on routinely collected clinical data, analyses were limited to measures already in use in the service. Consequently, anxiety and depression could not be examined separately, unlike in some previous studies, and psychological distress was instead assessed using the CORE-10. Similarly, Autism and ADHD were identified through unelicited, self-reported suspected or known diagnoses or ADHD medication use, rather than formal diagnostic assessment standardized screening measures, or a specific question. This approach may have introduced classification bias, as Autism or ADHD were often disclosed in the context of support needs. As a result, the number of patients identified as Autistic or ADHD was relatively small compared with prevalence estimates reported in ED populations (3–6), limiting statistical power and generalizability. This discrepancy is likely attributable to prior studies employing systematic screening measures across entire samples, rather than relying on spontaneous self-report. In addition, participants were classified as ADHD if they reported use of medications commonly prescribed for ADHD; however, it was not possible to confirm the indication for these prescriptions. For example, lisdexamfetamine is also approved for BED. Although, given the low number of patients with BED in this service (which is not commissioned for BED) this may have been less of a factor. However, some of these medications may have also been used off label for other conditions.

A new project, EDCRN, is establishing a UK-wide research network spanning child and adult ED services (18). As part of this project, a core dataset has been agreed across the network, to record ED symptoms, treatment, outcomes, demographics, risk factors and physical health markers. The dataset is being rolled out in participating ED services across the UK and includes the AQ-10 as a core measure and the ASRS as an optional measure. This will facilitate increased attention to Autism and ADHD in clinical ED settings and future studies to extend the findings presented here, which will allow us to better understand links between Autistic and ADHD traits and clinical measures in ED patients.

## 5 Conclusion

Autistic and ADHD patients with EDs may present to services with increased clinical complexity and elevated treatment needs, including greater ED psychopathology, psychological distress, and work and social functioning, particularly for those who are both Autistic *and* ADHD. Clinically, this highlights the importance of early identification and person-centred care. It also suggests that adaptations targeting Autistic and ADHD needs, such as sensory regulation, executive functioning support, and tailored psychological interventions are needed to improve engagement and treatment outcomes.

## Data Availability

The data analysed in this study is subject to the following licenses/restrictions: The data cannot be made available according to National Health Services guidance. Requests to access these datasets should be directed to KT, kate.tchanturia@kcl.ac.uk.
