# Supplementary material for: Time to notice neurodiversity in eating disorder services: a three-year real-world analysis of autism, ADHD, and AuDHD
Source: Front Psychiatry. 2026 Apr 10;17:1787957. doi: 10.3389/fpsyt.2026.1787957 (PMC13106426; doi:10.3389/fpsyt.2026.1787957)
Supplement: Supplementary file 1 [file Supplementaryfile1.docx]

Supplementary Material

# Appendix 1. Patient Intake Form, excluding CORE10, EDE-Q, AQ-10, and WSAS.

1. Trust ID (please enter in the format xx-xx-xx) *
2. Which service are you currently using*
   - SLaM Eating Disorders Outpatients Service
   - SLaM Eating Disorders Inpatient Service (Bethlem Royal Hospital)
   - SLaM Eating Disorders Day Services
   - SLaM Eating Disorders Enhanced Treatment Team (ETT)
3. Clinician Name (if known)
4. Timepoint
   - Assessment
   - Treatment Start
   - Mid Treatment
   - Treatment End
   - Follow Up 1
   - Follow Up 2
   - Other: ___________
5. Date of Birth*
6. Age*
7. Gender*
   - Female
   - Male
   - Non-binary
   - Other: ___________
8. Pro-noun*
   - She/Her
   - He/Him
   - They/Them
   - Other: ___________
9. Sexual orientation*
   - Heterosexual
   - Gay
   - Lesbian
   - Bisexual
   - Asexual
   - Unsure or prefer not to say
   - Other: ___________
10. Ethnicity*
    - Heterosexual
    - Gay
    - Lesbian
    - Bisexual
    - Asexual
    - Unsure or prefer not to say
    - Other: ___________
11. Is English your first language? *
    - Yes
    - No
12. What is your marital status? *
    - Married
    - Living together
    - Single
    - Divorced
    - Separated
    - Widowed
    - Other: ___________
13. What is your current employment status? *
    - Full time
    - Part time
    - Unemployed
    - Student
    - Retired
    - Sick leave
    - Housewife/husband
    - Other: ___________
14. What is the highest level of education you completed? *
    - No qualifications
    - O Level / GCSE
    - A Level / NVQ
    - Diploma / BTEC
    - University Degree
    - Postgraduate Degree
    - Other: ___________
15. How many years of education have you received? 
    (Include primary school, secondary school and any further education you have had.)
16. Have you had to take time off from school due to your eating disorder? *
    - Yes
    - No
17. Have you had to take time off from work due to your eating disorder? *
    - Yes
    - No
    - Not applicable
18. Who lives in your household with you? *
    - Parent(s)
    - Sibling(s)
    - Housemate(s)/friend(s)
    - Spouse
    - Partner
    - Parent(s) and sibling(s)
    - Parent(s), sibling(s), grandparent(s)
    - Live alone
    - Child/children
    - Spouse and child/children
    - Other: ___________
19. Do you own, rent or share your accommodation? *
    - Own
    - Rent
    - Share/live with family (e.g., with parents)
    - Council accommodation
    - Sheltered or temporary accommodation
    - Other: ___________
20. Has anyone in your family been diagnosed with Autism? *
    - Yes
    - No
21. Do you have any children? *
    - Yes
    - No
22. For how many years have you had an eating disorder? *
23. Have you had any professional help for eating disorders before? (choose one or more)

- Hospital admission(s)
- Day patient treatment
- Outpatient treatment
- No
- Other: ___________

1. Have you ever attempted suicide? *

- Yes
- No

1. Accessible information needs: Please identify if you need support in **verbal** communication *
   - No accessible support needs
   - Interpreter needed
   - Hearing loss or hearing aids used
   - Contact via text or email only
   - Other: ___________
2. Accessible information needs: Please identify if you need support in **visual** communication: *
   - No accessible support needs
   - Large font size
   - Contact via text or email only
   - Other: ___________
3. Please specify any other support needs
4. Do you currently have any mental health diagnoses other than your eating disorder? If yes, please give details below
5. Current medications and doses (if none, leave blank)
6. Current weight (in kg), approximate is fine
7. Current height (in cm), approximate is fine
8. Recently has your weight been
   - Increasing
   - Decreasing
   - Staying about the same
9. Lowest weight you have ever been? (in kg) Approximate is fine
10. What was your height at the time? (in cm)
11. Highest weight you have ever been? (in kg) Approximate is fine
12. What was your height at the time? (in cm)
13. Do you currently smoke tobacco?
    - Yes
    - No

**Table S1.** Missingness data for all cases, shown overall and separately for the four groups. Cells where *>*5% of data is missing are orange.

|  | Overall, % | AuDHD, % | Autism-only, % | ADHD-only, % | Neither, % |
| --- | --- | --- | --- | --- | --- |
| Age | 5.35 | 0.00 | 0.00 | 0.00 | 6.12 |
| Gender identity | 4.07 | 0.00 | 0.00 | 0.00 | 4.66 |
| Sexual orientation | 4.07 | 0.00 | 0.00 | 0.00 | 4.66 |
| Ethnicity | 4.15 | 0.00 | 0.00 | 0.00 | 4.75 |
| Level of Education | 4.07 | 0.00 | 0.00 | 0.00 | 4.66 |
| ED diagnosis | 20.93 | 21.88 | 33.33 | 18.52 | 20.57 |
| Service type | 0.00 | 0.00 | 0.00 | 0.00 | 0.00 |
| Weight trend | 6.63 | 3.13 | 2.22 | 1.23 | 7.31 |
| EDE-Q | 0.00 | 0.00 | 0.00 | 0.00 | 0.00 |
| AQ-10 | 0.00 | 0.00 | 0.00 | 0.00 | 0.00 |
| Family autism | 0.00 | 0.00 | 0.00 | 0.00 | 4.66 |
| ADHD meds | 0.00 | 0.00 | 0.00 | 0.00 | 0.00 |
| Verbal support | 14.38 | 12.50 | 24.44 | 13.58 | 14.08 |
| Visual support | 14.38 | 12.50 | 24.44 | 13.58 | 14.08 |
| Absence from work | 4.07 | 0.00 | 0.00 | 0.00 | 4.66 |
| Absence from school | 4.07 | 0.00 | 0.00 | 0.00 | 4.66 |
| Suicide attempt | 4.07 | 0.00 | 0.00 | 0.00 | 4.66 |
| Smoking | 4.47 | 0.00 | 0.00 | 0.00 | 5.12 |
| Alcohol | 4.63 | 0.00 | 0.00 | 0.00 | 5.30 |
| CORE10 | 0.00 | 0.00 | 0.00 | 0.00 | 0.00 |
| WSAS | 0.16 | 0.00 | 0.00 | 0.00 | 0.18 |

**Table S2.** Demographic characteristics, ED information (diagnosis, service type, and weight trend), and alcohol consumption for all cases, shown overall and separately for the four groups. Cells where *n*<5 are orange and where *n*=0 are red.

|  | Overall, % | AuDHD, % | Autism-only, % | ADHD-only, % | Neither, % | Effect size, *v* |
| --- | --- | --- | --- | --- | --- | --- |
| Gender^1^ | | | | | | 0.08 |
| *Female* | 88.91 | 90.62 | 80.00 | 83.95 | 89.63 |  |
| *Male* | 8.67 | 0.00 | 15.56 | 11.11 | 8.45 |  |
| *Non-binary* | 2.42 | 9.38 | 4.44 | 4.94 | 1.92 |  |
| Sexual orientation^2^ | | | | | | 0.11 |
| *Heterosexual* | 74.86 | 55.17 | 52.63 | 63.89 | 76.38 |  |
| *Bi/pansexual* | 15.78 | 24.14 | 26.32 | 27.78 | 14.05 |  |
| *Lesbian/gay* | 6.61 | 20.69 | 13.16 | 4.17 | 6.04 |  |
| *Asexual* | 2.11 | 0.00 | 7.89 | 2.78 | 1.87 |  |
| *Queer* | 0.64 | 0.00 | 0.00 | 1.39 | 0.62 |  |
| Ethnicity | | | | | | 0.05 |
| *White* | 74.81 | 68.75 | 71.11 | 77.78 | 75.51 |  |
| *Mixed* | 8.48 | 9.38 | 13.33 | 9.88 | 8.13 |  |
| *Black* | 7.98 | 6.25 | 8.89 | 6.17 | 8.13 |  |
| *Asian* | 6.63 | 9.38 | 6.67 | 6.17 | 6.58 |  |
| *Other* | 1.60 | 6.25 | 0.00 | 0.00 | 1.65 |  |
| Level of Education^3^ | | | | | | 0.08 |
| *Postgrad* | 17.15 | 37.50 | 6.67 | 12.35 | 17.35 |  |
| *Undergrad* | 30.98 | 28.13 | 33.33 | 33.33 | 30.78 |  |
| *A level/NVQ* | 21.15 | 6.25 | 26.67 | 23.46 | 21.19 |  |
| *Diploma/BTEC* | 11.66 | 9.38 | 13.33 | 12.35 | 11.60 |  |
| *O level/GCSE* | 11.91 | 9.38 | 11.11 | 11.11 | 12.08 |  |
| *No formal* | 2.91 | 3.13 | 4.44 | 0.00 | 3.07 |  |
| ED diagnosis^4^ | | | | | | 0.07 |
| *AN* | 32.54 | 33.33 | 33.33 | 29.69 | 32.71 |  |
| *BN* | 25.62 | 25.00 | 26.67 | 29.69 | 25.29 |  |
| *BED* | 4.34 | 4.17 | 0.00 | 4.69 | 4.47 |  |
| *ARFID* | 4.13 | 4.17 | 13.33 | 9.38 | 3.41 |  |
| *EDNOS* | 33.37 | 33.33 | 26.67 | 26.56 | 34.12 |  |
| Service type | | | | | | 0.04 |
| *Outpatient* | 93.13 | 96.88 | 93.33 | 96.30 | 92.78 |  |
| *Inpatient* | 3.91 | 3.13 | 6.67 | 2.47 | 3.93 |  |
| *Day* | 2.48 | 0.00 | 0.00 | 1.23 | 2.96 |  |
| *ETT* | 0.48 | 0.00 | 0.00 | 0.00 | 0.55 |  |
| Weight trend | | | | | | 0.04 |
| *Increasing* | 29.85 | 41.94 | 29.55 | 33.75 | 29.19 |  |
| *Unchanging* | 41.83 | 35.48 | 38.64 | 35.00 | 42.70 |  |
| *Decreasing* | 28.31 | 22.58 | 31.82 | 31.25 | 28.11 |  |
| Alcohol consumption | | | | | | 0.08 |
| *Never* | 28.89 | 43.75 | 35.56 | 43.21 | 25.59 |  |
| *Monthly/less* | 28.88 | 31.25 | 33.33 | 30.86 | 26.87 |  |
| *x2-4/month* | 25.54 | 18.75 | 22.22 | 14.81 | 25.32 |  |
| *x2-3/week* | 13.82 | 6.25 | 8.89 | 7.41 | 13.99 |  |
| *x4+/week* | 2.93 | 0.00 | 0.00 | 3.70 | 2.93 |  |

AN=Anorexia Nervosa, ARFID=Avoidant Restrictive Food Intake Disorder, BED=Binge Eating Disorder, BN=Bulimia Nervosa, EDNOS=Eating Disorder Not Otherwise Specified, *V*= Cramer’ V. ^1^*n*=1 prefer not to say; ^2^*n*=112 unsure or prefer not to say; ^3^*n=*102 other; ^4^*n*=22 did not have an ED and were being treated for EUPD, GAD, low mood, or emetophobia.

**Table S3.** Effect sizes (Cohen’s *f*) for pairwise group comparisons on age (years), EDE-Q, AQ-10, CORE10, and WSAS scores.

|  |  | AuDHD | Autism-only | ADHD-only | Neither |
| --- | --- | --- | --- | --- | --- |
| **Age** | **AuDHD** | X | .0549 | .0081 | .0402 |
|  | **Autism-only** | .0549 | X | .0592 | .0361 |
|  | **ADHD-only** | .0081 | .0529 | X | .0479 |
|  | **Neither** | .0402 | .0361 | .0479 | X |
| **EDE-Q** | **AuDHD** | X | .0120 | .0041 | .0323 |
|  | **Autism-only** | .0120 | X | .0196 | .0199 |
|  | **ADHD-only** | .0041 | .0196 | X | .0579 |
|  | **Neither** | .0323 | .0199 | .0579 | X |
| **AQ-10** | **AuDHD** | X | .0596 | .1482 | .2543 |
|  | **Autism-only** | .0596 | X | .0922 | .2091 |
|  | **ADHD-only** | .1482 | .0922 | X | .1273 |
|  | **Neither** | .2543 | .2091 | .1273 | X |
| **CORE10** | **AuDHD** | X | .0521 | .0474 | .0996 |
|  | **Autism-only** | .0521 | X | .0116 | .0382 |
|  | **ADHD-only** | .0474 | .0116 | X | .0691 |
|  | **Neither** | .0996 | .0382 | .0691 | X |
| **WSAS** | **AuDHD** | X | .0327 | .0338 | .1069 |
|  | **Autism-only** | .0327 | X | .0027 | .0764 |
|  | **ADHD-only** | .0338 | .0027 | X | .1053 |
|  | **Neither** | .1069 | .0764 | .1053 | X |

Figure S1. AQ-10 scores across the four groups (AuDHD = dark blue, Autism-only = orange, ADHD-only = green, Neither = light blue). Dotted red line indicates the cut-off for probable Autism. Error bars show standard deviation.

**Table S4.** Family history of Autism, Support needs (verbal and visual), absence from work or school due to ED, history of one or more suicide attempt, and for all cases, shown overall and separately for the four groups. Cells where *n*<5 are orange*.*

|  | Overall, % | AuDHD, % | Autism-only, % | ADHD-only, % | Neither, % | Effect size, *v* |
| --- | --- | --- | --- | --- | --- | --- |
| Family history of Autism | 25.90 | 75.00 | 73.33 | 33.33 | 21.76 | 0.30 |
| Verbal support needs | 17.89 | 37.50 | 31.11 | 17.28 | 16.82 | 0.11 |
| Visual support needs | 17.41 | 25.00 | 26.67 | 18.52 | 16.73 | 0.06 |
| Absence from work^1^ | 24.00 | 56.00 | 48.28 | 42.19 | 21.61 | 0.18 |
| Absence from school | 17.49 | 21.88 | 35.56 | 22.22 | 16.20 | 0.10 |
| Suicide attempt | 26.89 | 56.25 | 42.22 | 35.80 | 24.64 | 0.15 |
| Smoking | 14.13 | 6.25 | 20.00 | 19.75 | 13.68 | 0.07 |

*V*= Cramer’ V, ^1^*n*=225 not applicable.
